# Supplementary material for: Encoding of 3D head direction information in the human brain
Source: Hippocampus. 2018 Dec 18;29(7):619–29. doi: 10.1002/hipo.23060 (PMC6618148; doi:10.1002/hipo.23060)
Supplement: Supplementary file 1 — SUPPORING FIGURE S1 3D movement trajectories during scanning. Different colors represent different scanning sessions. [file HIPO-29-619-s001.docx]

**SUPPORTING INFORMATION**

**Encoding of 3D head direction information in the human brain**

Misun Kim, Eleanor A. Maguire


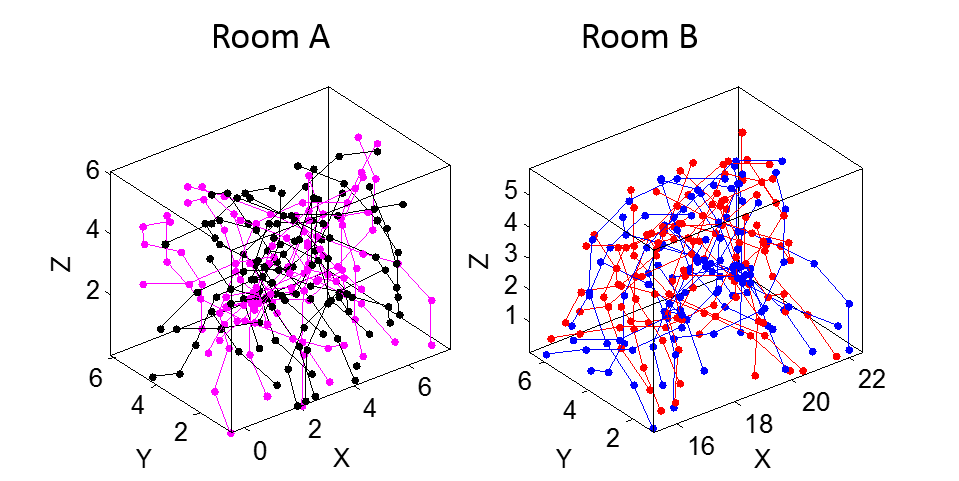


**SUPPORING FIGURE S1** 3D movement trajectories during scanning. Different colors represent different scanning sessions.
